# Fever

**Published:** 1855-01

**Authors:** 


Fever.—Be careful of your prognosis, when the nervous symptoms in fever preponderate at an early period of the attack, especially in the wealthy, who cannot bear wine so well as the poor. The more the secondary affections of fever are *anatomical*, the greater will be the use of stimulants; and conversely, the more they are neurotic, the less will be the use of stimulants.

When the tongue is dry and brown, head not very hot, respiration short, frequent, and irregular; and when added to these there is delirium, more or less violent, give twenty-five minims of chloroform in a draught, to be repeated in an hour. This may be repeated in a few hours if necessary, and if the pulse be watched, may be continued every second hour for some time. The inhalation of chloroform is useless in these cases, and may be followed by convulsive movements.—*Braithwaite's Retrospect.*

